# Predictors of Adherence to Antiretroviral Therapy among HIV/AIDS Patients in the Upper West Region of Ghana

**DOI:** 10.1155/2013/873939

**Published:** 2013-12-10

**Authors:** Christian Obirikorang, Peter Kuugemah Selleh, Jubilant Kwame Abledu, Chris Opoku Fofie

**Affiliations:** ^1^Department of Molecular Medicine, School of Medical Sciences, College of Health Sciences, Kwame Nkrumah University of Science and Technology (KNUST), Kumasi, Ghana; ^2^Department of Medical Laboratory Technology, Faculty of Allied Health, College of Health Sciences, Kwame Nkrumah University of Science and Technology (KNUST), Kumasi, Ghana; ^3^School of Veterinary Medicine, University of Ghana, Legon, Ghana; ^4^Ghana Health Service, Upper West Regional Hospital, Wa, Upper West Region, Ghana

## Abstract

*Background*. The effectiveness of ART interventions is only realized in maximal levels of adherence. A near perfect adherence level of >95% is required for the effective suppression of HIV/AIDS virus. The main objective of this study was to identify the sociodemographic and socioeconomic factors that facilitate adherence to antiretroviral therapy among HIV/AIDS patients. *Methods*. This descriptive cross-sectional study was conducted between March and May 2013 at the Upper West Regional Hospital, Wa. A total of 201 confirmed HIV 1 seropositive subjects (mean age 36.6 ± 9.9 years) receiving antiretroviral therapy were interviewed using a structured questionnaire. The collected data was analyzed using GraphPad Prism version 5. A *P* value of <0.05 was considered statistically significant for all statistical analyses. *Results*. Overall lifetime adherence was found to be 62.2% while medication adherence in the last six months, last three months, last month, and last week were 73.6%, 87.1%, 91.0%, and 86.0%, respectively. The study revealed a positive association between adherence to ART and immunological success, with nonadherence increasing the risk (OR (95% CI): 9.2 (3.2–26.9)) of immunological failure. Univariate logistic regression analysis of the data showed that other ailments and side effects of drug were negatively associated with adherence to ART whereas self-perceived wellness, family support, and regular followup were positively associated with adherence to ART. *Conclusion*. Regular attendance at followup and family support are vital factors for 100% lifetime medication adherence. Effective counseling sessions on adherence for patients on antiretroviral therapy are paramount for the realization of the purpose of antiretroviral therapy programmes in Ghana.

## 1. Introduction

The effect of HIV infection at the individual level is the continued breakdown of the immune system of the host which ultimately results in the onset of AIDS. All infected persons are at risk of illness and death from opportunistic infections and neoplastic complications [1]. Infection to noninfected individuals with HIV occurs mainly through the exposure to biological fluids, especially semen and blood, of the infected individuals. Globally, the principal route of transmission is unprotected heterosexual intercourse (>75%). This accounts for the increasing number of women being affected worldwide. Homosexual intercourse is the second commonest route of transmission [2].

Sub-Saharan Africa bears the greatest burden with more than two-thirds (68%) of all persons infected with HIV. An estimated 1.8 million adults and children became infected with the disease in Sub-Saharan Africa. It is recorded that out of 260,000 child deaths that occurred globally from HIV/AIDS in 2009, 88% occurred in Sub-Saharan Africa [3].

The HIV/AIDS epidemic in Ghana continues to be a generalized epidemic with a prevalence of more than 1% in the general population. Promising developments have been seen in recent years in global efforts to address the AIDS epidemic, including increased access to effective treatment and prevention programmes [4].

The number of HIV patients receiving ART in Ghana increased more than 200-fold from 197 in 2003 to over 45,000 in 2010. Some regions report ART enrollment lower than their percent share of number of HIV infected persons in the country [5]. The world Health Organization recommendations on the use of ART in resource-limited settings recognize the critical role of adherence in order to achieve clinical and pragmatic success. Good adherence to ART is necessary to achieve the best antivirological response, lower the risk that drug resistance will develop, and reduce morbidity [6].

Combination therapies of ARV drugs are the treatment of choice in HIV, and nonadherence is a major, if not the most important, factor in treatment failure and the development of resistance. 100% medication adherence is paramount for the effective management of HIV [2] and provision of free treatment without adequate patient preparation and adherence support may compromise the success of ART scale-up programmes [7]. A major concern with scaling up of antiretroviral therapy (ART) in resource-limited settings is the emergence of drug resistant viral strains due to suboptimal adherence and the transmission of these resistant viral strains in the population [7].

In view of the changing trend in prevalence of HIV in Ghana and the lack of data surrounding medication adherence in this population, this study therefore proposed to assess the level of and validate (using CD4 results) self-reported adherence and its predictors among patients attending the HIV Clinic of Upper West Regional Hospital, Wa.

## 2. Materials and Methods

This descriptive cross-sectional study was conducted at the Upper West Regional Hospital situated in the southern part of the capital Wa. It is the only specialized referral hospital in the region and the HIV unit currently provides service to over 1600 registered HIV/AIDS patients on antiretroviral drugs. The study was conducted between the months of March and May 2013. A total of two hundred and one (201) confirmed HIV 1 seropositive subjects receiving antiretroviral therapy were interviewed using a structured questionnaire with both open and close ended questions. Primary data was also obtained from their medical records after obtaining permission from the health facility administrators and consent from patients. The Committee on Human Research, Publications and Ethics, School of Medical Sciences, Kwame Nkrumah University of Science and Technology, gave ethical approval for the study to be conducted. Confidentiality, anonymity, and privacy were guaranteed.

### 2.1. Data Collection

A structured questionnaire which was developed from different literatures was used for the data collection. The dependent variable was adherence to highly active antiretroviral therapy (HAART) among PLWHA. The independent variables were sociodemographic (age, sex, weight, level of education, occupation, marital status, and family type), socioeconomic variables (income), psychosocial (social support, active substance and alcohol use, disclosure of HIV serostatus, and perception of well-being), disease characteristics (duration of HIV infection), regimen related variables (types of ART, dietary related demands/restriction, and side effect), CD4 at diagnosis and current value, followups, adherence to treatment information and symptoms associated with treatment.

Many researchers who have conducted studies in this area found that there is no existing gold standard by which adherence can be quantified and many predictors have been reported to influence it. The study therefore chose five measurement tools to quantify adherence from self-recalled report data collected from participants at exit face-to-face interviews:

- lifetime self-recall adherence,
- last 6 months' self-recall adherence,
- last 3 months' self-recall adherence,
- last month's self-recall adherence,
- last week's self-recall adherence.

Participants were asked if they had ever missed medication in their lifetime beginning from the time s/he was put on antiretroviral therapy. Self-reported adherence was classified as “adherent” when not a single dose was missed or nonadherent if the patient admitted having missed at least one dose. They were asked about adherence to medication since initiation of ART as listed above. This means that patients' memory of medicine intake was likely to be good.

However, in such face-to-face interviews patients might feel ashamed to report missed medications. Hence participants were assured of confidentiality for their response and they were made aware that, even though medically precarious, every individual could miss medication for one reason or the other.

### 2.2. Data Management and Analysis

Data collected was sorted, coded, and entered into an Excel spreadsheet for analysis using GraphPad Prism for Windows version 5.0 (GraphPad Software, San Diego, CA, USA). Descriptive statistics such as mean, frequencies, and percentages were used to summarize the data. Overall lifetime adherence to medication was determined from the data and predictors determined. Analysis of contingency tables was done and Fisher's exact test and the chi-square test were used where necessary to compare proportions. Logistic regression analysis was used to determine the relationships between adherence and other variables (level of significance, *P* < 0.05).

## 3. Results

The cross-sectional study included 201 individuals diagnosed with HIV, who had since been receiving ART at the Regional Hospital at Wa in the Upper West Region of Ghana between 2003 and 2012. The patients' demographic and clinical data are presented in Tables 1 and 2. The age range of patients was 20–74 years with the mean ± standard deviation of the age being 36.6 ± 9.9 years. The males (42.3 ± 11.0 years) were significantly older (*P* < 0.0001) than the females (35.1 ± 9.0 years) although the latter constituted the majority (79.1%) of the population. Whereas only 4.5% had attained higher (tertiary) education, 52.2% were married and as many as 52.7% were living with their nuclear family. Monthly income not exceeding GH*¢* 100.00 was earned by as many as 92.0% of the patients.

Most patients (80.1%) were diagnosed during the last four years (i.e., 2008–2012), with 158 patients attending regular followup, that is, attending greater than 90% of 3 monthly appointments. The overall lifetime medication adherence (i.e., taking all medications everyday as prescribed) since initiating ART was found to be 62.2% (*n* = 125). Of the respondents who had participated in the study, as many as 73.6%, 87.1%, 91.0%, and 86.0% had adhered to medication in the last six months, last three months, last month, and last week, respectively (Table 1).

Of those who enumerated reasons for missing ART, 46.1% attributed it to forgetfulness and 7.2% said they had no food, among other reasons as shown in Table 2. The median rise in CD4 cell count from baseline to now was 119 cells/mm^3^, with a proportion of 21.3% meeting the criteria for immunological failure (i.e., a drop in CD4 cell count to pretreatment levels or <100 cells/mm^3^) (Table 1). Besides that, about 15.1% of subjects had a CD4 count of <100 cells/mm^3^ at diagnosis and 0.6% had a current CD4 count of <100 cells/mm^3^.

All except four subjects were on NNRTI and NRTI or in combination with septrin prophylaxis. The four exceptional subjects were on septrin prophylaxis only. As many as 41.8% were on nevirapine-based combination therapy and 32.8% on efavirenz-based combination therapy as shown in Table 2.

Univariate analysis of individual patient factors associated with medication adherence is recorded in Table 3. Gender, education, marital status, type of family, disclosure of status to other persons, time since diagnosis was made, time since ART was initiated, perceived difficulty of drug regimen, and food restrictions were not associated (*P* > 0.05) with medication adherence. Although not statistically significant, positive trends for medication adherence were seen for increasing age (*P* = 0.3401) and monthly income (*P* = 0.6238). However, as shown in the univariate analysis (Table 3), regular followup (OR: 10.4; CI: 4.6–23.6; *P* < 0.0001), perceiving oneself as very healthy (OR: 9.0; CI: 3.4–23.9; *P* < 0.0001), and family support (OR: 1.9; CI: 1.1–3.5; *P* = 0.0401) were the most significant positive factors associated with increased medication adherence. On the other hand, adherence to medication was significantly reduced in patients who suffered other ailments (OR: 0.2; CI: 0.1–0.5; *P* = 0.0004) and those who suffered side effects of drug (OR: 0.2; CI: 0.1–0.4; *P* < 0.0001). Some of the patients perceived their ART regimen as simple (*n* = 94) and moderate (*n* = 100). Although self-perceived well-being was generally high (normal (*n* = 143) and very healthy (*n* = 54)), 17.4% of patients reported having side effects of the drugs, 14.4% presented with some other ailments, whilst about 91.5% were currently placed on food/substance restriction. Also, some participants (79.0%) reported having disclosed their HIV status to at least one person and 69.2% were satisfied by their family support.

To construct a predictive model of the determinants of adherence, the significant independent factors were entered simultaneously into a multivariate logistic regression model. The results are presented in Table 3. All other factors but family support (OR: 0.9; CI: 0.40–1.97; *P* = 0.7683) retained their significance. In this adjusted model, patients who suffered other ailments (OR: 0.3; CI: 0.10–0.81; *P* = 0.0178) improved slightly on adherence to medication although the association maintained its negativity; side effects of drug (OR: 0.2; CI: 0.1–0.6; *P* = 0.0016) maintained a constant negative association with adherence as was in the univariate analysis, while regular followups (OR: 6.9; CI: 2.8–17.0; *P* < 0.0001) and perceiving oneself as very healthy (OR: 4.2; CI: 1.5–12.1; *P* = 0.0078) slightly decrease adherence (as compared to the univariate analysis) albeit maintaining their positive association with medication adherence.

**Figure 1:**
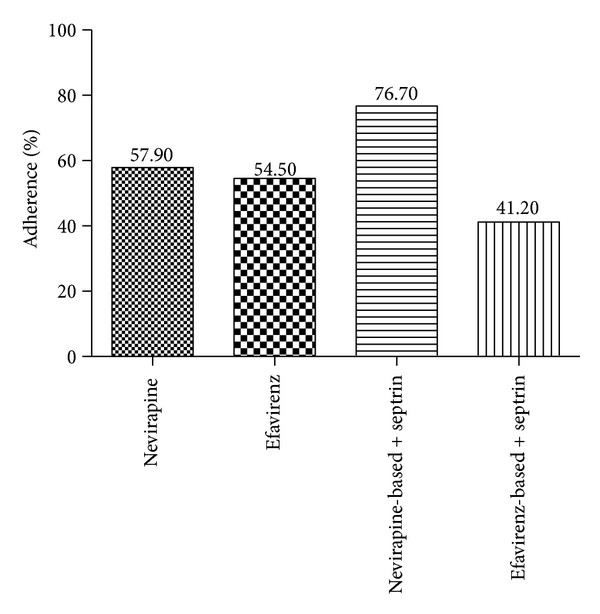
Adherence levels based on patient's antiretroviral therapy combinations.

Figure 1 shows the adherence levels based on patient's ART combinations. With the exception of few respondents who were taking septrin alone (2.0%), all other participants were taking the three combinations of ART from the nucleotide and nucleoside reverse transcriptase inhibitors and the nonnucleoside reverse transcriptase inhibitors classes. The majority of the respondents (25.9%) were taking stavudine (d4t)/lamivudine (3TC)/nevirapine (NVP) combination. Figure 1 presents the adherence pattern based on the different combinations of ART the respondents were taking. The majority of nonadherent participants were on efavirenz based combination therapy and most adhering respondents were on nevirapine-based combination therapy. Patients taking a combination of efavirenz and septrin had comparatively reduced adherence as compare to those on efavirenz only.

## 4. Discussion

Antiretroviral therapy adherence levels of ≥95% optimize outcomes and minimize viral resistance [6]. The overall lifetime adherence (i.e., taking all medications everyday as prescribed and abiding by the food/substance restrictions) since initiating ART therapy was found to be suboptimal (62.2%). This adherence level is similar to a study report in Bangalore, India [2], where overall lifetime adherence was 60.4%. On the other hand, high proportions of the study respondents had not missed medication in the last three months, last month and last week (87.1%, 91.0%, and 86%, resp.). The overall lifetime adherence level of the present study is higher than the adherence level in the developed countries (55%), as shown in a meta-analysis of adherence to ART in both developed and developing countries [8]. The many study reviews conducted in this field in Africa revealed an adherence level similar or higher than the adherence level in the developed countries. This review finding is against the initial fears raised prior to ART initiation in the African continent and has shown that a higher adherence level can be achieved in Africa despite limited resources.

There are concerns that patients on ART have the tendency to be nonadherent once they feel physically better [9]. The current study however revealed, on the contrary, that respondents with high perception of well being were more adherent to ART therapy. This could be due to respondents' realization of the usefulness of the medication and the fact that they were heeding the counseling instructions offered prior to ART initiation. This deduction is buttressed by a study conducted by Mills et al. [8] on “Meta-analysis of ART adherence in both developed and developing countries.” There was no significant relationship (*P* > 0.05) in levels of adherence in relation to the duration a patient had been taking ART as well as food/substance restriction as shown in Tables 1 and 3, respectively.

In univariate analysis, regular followup, family support, and perception of well being were positively associated with adherence to medication as shown in Table 3. Traditionally, before the infiltration of Western culture, Africans were identified by the extended family system which has its own benefits and demerits. The results of our study show no statistically significant relationship between family type and adherence to antiretroviral therapy. However patients who had family support adhered to the antiretroviral therapy (*P* = 0.0401). This is consistent with a study report from Southwest Ethiopia where participants who had family support were two times more likely to adhere than respondents on the other side of the coin [10]. This study finding is also in support of study review findings in Kenya where family support was characteristic of patients who achieved optimal adherence [6].

The majority of patients (79%) in our study had disclosed their status to at least one relative, a reflection that the study site is complying with the national guidelines for managing HIV/AIDS patients on ART. Besides that, about 69.2% of study participants reported they had support from family members in the form of emotional/psychological, physical, and food provision. These could be the promoters of optimal adherence among these participants as family members could remind them to take the ART, give comfort, and provide food to take ART with. The findings of this study suggest that a poor psychological state could be associated with poor adherence. Other studies have also shown a significant association between poor psychological states and nonadherence [11].

Moreover, regular followup is another positive predictor of adherence to ART per the results of our study (*P* < 0.0001). This finding is consistent with the numerous studies from both developed and developing countries [2, 8]. Of those who reported regular followup, 73.4% were adherent. This could be due to constant counseling, patients being able to express concerns about medication and their health, and receiving medications thus avoiding running out of drugs.

The study results demonstrated positive correlation between adherence to ART and immunological success; nonadherent subjects were nine times (OR (95% CI): 9.2 (3.2–26.9)) at risk of progressing to immunological failure (that is, a drop in CD4 cell count to pretreatment value or <100 cells/mm^3^ whilst on treatment for a period of time) compared to their adherent counterparts as depicted in Table 1. This immunological finding indicates that body systems of respondents in this category responded positively to the ARV. A greater proportion of these participants also had good perception of well-being. This finding is similar to a study report from the resource-limited setting of Southwest Ethiopia [10]. Our study revealed that about 21.3% of respondents met the criterion for immunological failure where most of these persons constituted the nonadherent group of this study. Our study found no statistically significant relationship between gender and adherence to ART. However, females constituted a greater proportion (79.1%) of the study participants; “this is a reflection of the gender distribution of HIV/AIDS” in Ghana as revealed by other studies [12]. It can be argued that females are more aware of their status and have access to ART therapy. This is consistent with study reports from most resource-limited settings like the neighboring Togo where out of 99 participants in a study, 76.8% were females [13].

The majority of the participants who did not adhere to ART provided varied motives for their defaults. A large proportion (46.1%) of people in this subset cited forgetfulness as reasons for missing therapy and 42.1% said they missed medication due to the fact that they ran out of drugs. Of those who did not adhere, 9.2% said they had no food to take with the drug and 15.8% said they were away from home as reasons for missing ART treatment. These findings were similar to works done in South West Ethiopia [10], India [7], Kenya [6], Zambia [14], and South Africa [11].

Some of the respondents (14.8%) suffered other ailments and adherence to ART was significantly reduced in this category of respondents. The most common ailments mentioned were coughing, hernia, diabetes, high blood pressure, chest pains, ulcer, rashes, general weakness, and skin itching. Our study found that adherence was negatively affected (*P* < 0.001) in respondents who suffered side effects of the drugs (17.4%). This finding is consistent with the study report by [13].

All study participants were on the standard first-line regimen proposed by WHO (2NRTI+1NNRTI) and all patients were managed on the three combinations as found in other study reports in Africa [12, 13]. The study found no significant relationship between type of ART combination and adherence although the majority of nonadherent participants were on efavirenz based combination therapy. Some of HIV/AIDS patients on ART experience side effects. However, side effects were cited by most respondents on efavirenz-based combination therapy. Almost all participants on efavirenz-based combination therapy in this class cited sleepiness and/or dizziness as side effects experienced. Other side effects mentioned by participants include headache, cold, general weakness, and excessive urination. Adherence to ART was negatively affected in these patients who experienced side effects. They skipped medication to avoid side effects and this could explain why the majority of nonadherent participants in this study are those on efavirenz-based combination therapy. This outcome is consistent with studies done in other African countries [6, 10].

## 5. Conclusion

The findings of the study show that the lifetime adherence was suboptimal. Factors such as regular followup and psychological and physical support were found to be positive promoters of ART adherence. However, other ailments and side effects of the drugs had a negative association with adherence to ART.

## Figures and Tables

**Table 1:** Patient demographics relative to medication adherence.

Parameter	Total (%)	No (%)	Yes (%)	*P* value
Missed medications in lifetime				
Ever missed medications since starting ART	201 (100)	125 (62.2)	76 (37.8)	
Missed medications in the last 6 months	201 (100)	148 (73.6)	53 (26.4)	
Missed medications in the last 3 months	201 (100)	175 (87.1)	26 (12.9)	
Missed medications in the last month	201 (100)	183 (91.0)	18 (9.0)	
Missed medications in the last week	201 (100)	172 (86.0)	28 (14.0)	
Age				
20–30	64 (32.5)	38 (59.4)	26 (40.6)	
31–40	83 (42.1)	53 (63.9)	30 (36.1)	0.3401
>40	50 (25.4)	34 (68.0)	16 (32.0)	
Sex				
Male	42 (20.9)	24 (57.1)	18 (42.9)	0.4774
Female	159 (79.1)	101 (63.5)	58 (36.5)	
Marital status				
Married	105 (52.2)	62 (59.0)	43 (41.0)	
Widowed	45 (22.4)	28 (62.2)	17 (37.8)	0.7060
Divorced	30 (14.9)	21 (70.0)	9 (30.0)	
Single	21 (10.4)	14 (66.7)	7 (33.3)	
Education				
No education	102 (50.7)	62 (60.8)	40 (39.2)	
Primary	31 (15.4)	21 (67.4)	10 (32.3)	
JHS/middle school	49 (24.4)	30 (61.2)	19 (38.8)	0.7223
SHS	10 (5.0)	5 (50.0)	5 (50.0)	
Tertiary	9 (4.5)	7 (77.8)	2 (22.2)	
Family type				
Nuclear	106 (52.7)	59 (55.7)	47 (44.3)	
Extended	67 (33.3)	46 (68.7)	21 (31.3)	0.1268
Living alone	28 (13.9)	20 (71.4)	8 (28.6)	
Monthly income				
≤100	104 (92.0)	71 (68.3)	33 (31.7)	
101–500	7 (6.2)	5 (71.4)	2 (28.6)	0.6238
>500	2 (1.8)	2 (100.0)	0 (0.0)	
Time since ART (years)				
<1	7 (3.5)	1 (14.3)	6 (85.7)	
1–4	163 (81.1)	66 (40.5)	97 (58.4)	0.2058
5+	31 (15.4)	9 (29.0)	22 (71.0)	
Time since diagnosis (years)				
<1	3 (1.5)	0 (0.0)	3 (100.0)	
1–4	158 (78.6)	62 (39.2)	96 (60.8)	0.3507
5+	40 (19.9)	14 (35.0)	26 (65.0)	
Immunological success				
Yes	96 (78.7)	66 (68.8)	30 (31.3)	0.001*
No*	26 (21.3)	5 (19.2)	21 (80.8)	

Data are presented as proportions. Proportions are compared using Fisher's exact test and the chi-square test where applicable. *(OR: 9.2; 95% CI: 3.2–26.9).

**Table 2:** Patient antiretroviral therapy and disease characteristics.

Parameter	Frequency/percentage
CD4 at diagnosis (cells/mm^3^)	
<100	26 (15.1)
101–200	35 (20.3)
201–300	56 (32.6)
>300	55 (32.0)
Current ART regimen	
Nevirapine based	84 (41.8)
Efavirenz based	66 (32.8)
Nevirapine based + septrin	30 (14.9)
Efavirenz based + septrin	17 (8.5)
Septrin	4 (2.0)
Current CD4 (cells/mm^3^)	
<100	1 (0.6)
101–200	4 (2.4)
201–300	21 (12.5)
>300	142 (84.5)
Reason for missing ART	
Forgetfulness	35 (46.1)
No food to take with	7 (9.2)
Away from home	12 (15.8)
Felt sick	2 (2.6)
Busy	3 (3.9)
Run out of drugs	32 (42.1)

Data are presented as frequency and percentage. Data may not add up to 201 due to missing data.

**Table 3:** Logistic regression of patient characteristics associated with medication adherence.

Characteristic	Total	Nonadherent	Adherent	OR (95% CI)	*P* value	aOR (95% CI)	*P* value
Food restriction							
Yes	183 (91.5)	71 (38.8)	112 (61.2)	0.7 (0.2–1.9)	0.3896		
Other ailments							
Yes	29 (14.4)	20 (69.0)	9 (31.0)	0.2 (0.1–0.5)	**0.0004**	0.3 (0.1–0.8)	**0.0178**
No*	167 (85.2)	54 (32.3)	113 (67.7)	1		1	
Side effects							
Yes	35 (17.4)	26 (74.2)	9 (25.7)	0.2 (0.1–0.4)	**<0.0001**	0.2 (0.1–0.6)	**0.0016**
No*	166 (82.6)	50 (30.1)	116 (69.9)	1		1	
Perceived difficulty of drug regimen							
Simple*	94 (46.8)	41 (43.6)	53 (56.4)	1			
Moderate	100 (49.8)	31 (31.0)	69 (69.0)	1.7 (1.0–3.1)	0.0701		
Difficult	7 (3.4)	4 (57.1)	3 (42.9)	0.6 (0.1–2.7)	0.4916		
Perceived well-being							
Normal*	143 (71.5)	69 (48.3)	74 (51.7)	1		1	
Very healthy	54 (27.0)	5 (9.3)	49 (90.7)	9.0 (3.4–23.9)	**<0.0001**	4.2 (1.5–12.1)	**0.0078**
Sick	3 (1.5)	2 (66.7)	1 (33.3)	0.5 (0.1–5.2)	0.5299		
Disclosure							
Yes	158 (79.0)	63 (39.9)	95 (60.1)	1.5 (0.7–3.1)	0.2915		
No*	42 (21.0)	13 (31.0)	29 (69.0)	1			
Family support							
Yes	139 (69.2)	46 (33.1)	93 (66.9)	1.9 (1.1–3.5)	**0.0401**	0.9 (0.4–2.0)	**0.7683**
No*	62 (30.8)	30 (48.4)	32 (51.6)	1		1	
Followup							
Regular	158 (78.6)	42 (26.6)	116 (73.4)	10.4 (4.6–23.6)	**<0.0001**	6.9 (2.8–17.0)	**<0.0001**
Irregular*	43 (21.4)	34 (79.1)	9 (20.9)	1		1	

*Reference. Data are presented as proportions. Regular followup implies more than 90% attendance at 3 monthly appointments. OR: odds ratio, aOR: adjusted odds ratio, CI: confidence interval. Data may not add up to 201 due to missing data. Other ailments (0: no; 1: yes); perception of well-being (1: normal; 2: very healthy; 3: sick); side effects (0: no; 1: yes); support from family (0: no; 1: yes); followup (0: irregular; 1: regular-0) Perceived difficulty of drug regimen (simple: 1; moderate: 2; difficult: 3) Disclosure (yes: 1; no: 0).
